# 9.N. Workshop: It Takes Two To Improve European Child Health (Care)

**DOI:** 10.1093/eurpub/ckac129.600

**Published:** 2022-10-25

**Authors:** Organised by: EUPHA-CAPH, Chair person: Danielle Jansen (EUPHA-CAPH)

## Abstract

Many societal developments have an impact on health care in general and on child health care, in particular. Internationally challenges include an aging population, more cultural diversity and a rising prevalence of chronic diseases among children and adolescents, revealing potential risks for child health care. A strong focus must be placed on prevention strategies that are effective, sustainable and equitable. Planning and implementing prevention strategies may require shifts in the organization of care, such as the forging and strengthening of interdisciplinary and intersectoral partnerships within a country. A promising example of such partnerships is the collaboration of public health with paediatrics. Both professional groups acknowledge the call for greater integration since prevention strategies can only be achieved and sustained by working together.

The objectives of this workshop are to:

• Provide a selective overview of three partnerships and plans for cooperation between public health and paediatrics.

• To exchange experiences and possibilities with the audience to pave the way for further successful partnerships.

In this workshop, we outline the partnership and plans for cooperation between public health and paediatrics in three European countries: Netherlands, Finland and Switzerland. In the first presentation, Danielle Jansen and Károly Illy will share the new vision towards the year 2030 of the Dutch Paediatric Society in which building blocks are presented to guarantee accessible, high-quality, timely and effective care for every child. One of the building blocks to be highlighted is the interprofessional collaboration between paediatricians and public health professionals. In the second presentation, Julia Dratva and Susanne Stronski from Switzerland will present a shared paediatric and public health vision of a digital child health booklet. The digital booklet will empower parents and adolescents, provide access to personal health irrespective of place or time, improve sharing health information among care professionals, thus ensuring continuity of care and limiting redundancy of investigations and in addition, and provide data for public health research and monitoring. Challenges and solutions will be shared with the audience. In the third presentation, Silja Kosola from Finland will present a Finnish vision for school health care where professionals trained in public health and medicine collaborate with each other as well as with teachers for the benefit of children and adolescents. This multidisciplinary collaboration across governing bodies is especially important as Finland undergoes a national reform of social and health care services. After the three presentations, we engage the audience by asking for their experiences and sharing the examples of collaborations between public health and paediatrics, as well as barriers and facilitators. At the end of the workshop, we would like to summarize the results of the workshop in an overview of preliminary best practices.

**Key messages:**

• Global climatic, societal and politic developments reveal potential risks for child health cand health care, which must be countered effectively, sustainably and equitably.

• Greater integration of prevention across sectors is elemental and can be achieved through interprofessional partnerships.

